# Submacular integration of hESC-RPE monolayer xenografts in a surgical non-human primate model

**DOI:** 10.1186/s13287-021-02395-6

**Published:** 2021-07-27

**Authors:** Zengping Liu, Tanja Ilmarinen, Gavin S. W. Tan, Heidi Hongisto, Edmund Y. M. Wong, Andrew S. H. Tsai, Sami Al-Nawaiseh, Graham E. Holder, Xinyi Su, Veluchamy Amutha Barathi, Heli Skottman, Boris V. Stanzel

**Affiliations:** 1 Department of Ophthalmology, Yong Loo Lin School of Medicine, National University of Singapore, Singapore, Singapore; 2 Institute of Molecular and Cell Biology (IMCB), Agency for Science, Technology and Research (A*STAR), Singapore, Singapore; 3 Singapore Eye Research Institute, Singapore, Singapore; 4 Faculty of Medicine and Health Technology, Tampere University, Arvo Ylpön katu 34, 33520 Tampere, Finland; 5 Singapore National Eye Centre, Singapore, Singapore; 6 Ophthalmology Academic Clinical Research Program, DUKE-NUS Graduate Medical School, Singapore, Singapore; 7 Department of Ophthalmology, Institute of Clinical Medicine, University of Eastern Finland, Kuopio, Finland; 8 Eye Clinic Sulzbach, Knappschaft Hospital Saar, An der Klinik 10, Sulzbach, 66280 Saar Germany; 9 Institute of Ophthalmology, University College London, London, United Kingdom; 10 Department of Ophthalmology, National University Hospital, Singapore, Singapore; 11 Department of Ophthalmology, University of Bonn, Bonn, Germany

**Keywords:** Retinal pigmented epithelium, Pluripotent stem cells, Cellular therapy, Cell transplantation, Non-human primate model

## Abstract

**Background:**

Human pluripotent stem cells (hPSCs) provide a promising cell source for retinal cell replacement therapy but often lack standardized cell production and live-cell shipment logistics as well as rigorous analyses of surgical procedures for cell transplantation in the delicate macula area. We have previously established a xeno- and feeder cell-free production system for hPSC differentiated retinal pigment epithelial (RPE) cells, and herein, a novel immunosuppressed non-human primate (NHP) model with a disrupted ocular immune privilege is presented for transplanting human embryonic stem cell (hESC)-derived RPE on a scaffold, and the safety and submacular graft integration are assessed. Furthermore, the feasibility of intercontinental shipment of live hESC-RPE is examined.

**Methods:**

Cynomolgus monkeys were systemically immunosuppressed and implanted with a hESC-RPE monolayer on a permeable polyester-terephthalate (PET) scaffold. Microscope-integrated intraoperative optical coherence tomography (miOCT)-guided surgery, postoperative follow-up incorporated scanning laser ophthalmoscopy, spectral domain (SD-) OCT, and full-field electroretinography (ERG) were used as outcome measures. In addition, histology was performed after a 28-day follow-up.

**Results:**

Intercontinental cell shipment, which took >30 h from the manufacturing to the transplantation site, did not alter the hESC-RPE quality. The submacular hESC-RPE xenotransplantation was performed in 11 macaques. The miOCT typically revealed foveal disruption. ERG showed amplitude and peak time preservation in cases with favorable surgical outcomes. Histology confirmed photoreceptor preservation above the grafts and in vivo phagocytosis by hESC-RPE, albeit evidence of cytoplasmic redistribution of opsin in photoreceptors and glia hypertrophy. The immunosuppression protocol efficiently suppressed retinal T cell infiltration and microglia activation.

**Conclusion:**

These results suggest both structural and functional submacular integrations of hESC-RPE xenografts. It is anticipated that surgical technique refinement will further improve the engraftment of macular cell therapeutics with significant translational relevance to improve future clinical trials.

**Supplementary Information:**

The online version contains supplementary material available at 10.1186/s13287-021-02395-6.

## Background

The eye is a frontline target for regenerative medicine utilizing human pluripotent stem cell (hPSC)-derived cells. This reflects the high demand for novel treatments for common blinding diseases, particularly age-related macular degeneration (AMD), and the ability for non-invasive graft monitoring. AMD pathogenesis is characterized by local dysfunction and subsequent degeneration of the retinal pigment epithelium (RPE), together with photoreceptor death, causing irreversible central visual loss [1]. Delivery of novel retinal therapeutic agents, particularly RPE cell therapy, has received considerable interest since hPSC-RPE were introduced for clinical applications since the feasibility of RPE transplantation in non-human primates (NHP) in the 1980s [2]. Recently, several clinical trials have explored the safety and feasibility of submacular delivery of either human embryonic stem cell (hESC) or human induced PSC (hiPSC)-derived RPE with the injection of cell suspensions [3–5], or with RPE monolayers with or without an artificial supportive scaffold [6–8].

However, several critical questions remain. First, since unsupported RPE transplants have integration-related challenges [3, 8], several carrier substrates have been proposed, including biostable parylene [7], polyester-terephthalate (PET) [6], and biodegradable poly(lactic-co-glycolic acid) (PLGA) scaffolds [9]. A scaffold facilitates subretinal graft integration but adds complexity to the surgical procedure. The human retina has the macula lutea, a highly specialized anatomic location at the posterior pole of the eye, shared only by diurnal primates. As the macula is the target site for RPE transplantation in AMD, it is essential to study surgical protocols and their implications in a NHP model.

Second, it is essential to study the risk of graft rejection impacts both safety and efficacy in the NHP model. Previously, hPSC-RPE cells have been transplanted into NHP with the intact healthy host RPE [10–12]. That approach may reduce the immune response to the grafts as the immune privilege of the subretinal space is not compromised [13, 14]; transplantation of hPSC-RPE on supportive scaffolds into a NHP with disrupted ocular immune privilege has not previously been reported.

Third, approaches using a bio-engineered monolayer graft also face the challenge of delivering the live culture grafts from the site of manufacture to the site of transplantation. Currently, protocols for cryopreservation of RPE cells as mature, postmitotic monolayer sheets are lacking. In clinical trials, an 8-h delivery window has been reported [6], making shipment to clinical centers distant from the cell production site demanding.

Herein, the safety and efficacy of submacular transplantation of hESC-RPE monolayer xenografts were analyzed in a NHP model with a mechanically disrupted blood-retinal barrier. This setting allowed evaluation of the role of submacular surgical protocols and a systemic immunosuppression regime. The feasibility of long-distance shipment of live hESC-RPE monolayers was also assessed.

## Methods

### Human ESC differentiation to RPE

Tampere University has National Supervisory Authority for Welfare and Health (Dnro 1426/32/300/05) approval to conduct research on human embryos. The institute also has supportive statements of the Ethical Committee of the Pirkanmaa Hospital District to derive, culture, and differentiate hESC lines (Skottman/R05116). No new cell lines were derived for this study.

Human ESC line Regea08/01 7[15] was cultured and differentiated to RPE as described previously [16]. Briefly, hESCs were detached with TrypLE™ Select into suspension in xeno-free differentiation medium (XF-Ko-SR) containing KnockOut DMEM supplemented with 15% KnockOut SR XenoFree CTS, 2 mM GlutaMAX, 0.1 mM 2-mercaptoethanol, 1% MEM non-essential amino acids solution, and 50 U/ml penicillin-streptomycin (all from Thermo Fisher Scientific). Embryoid body (EB) formation was induced overnight by the addition of 10 μM blebbistatin (Sigma-Aldrich, Saint Louis, MO, USA). Following EB formation, a 3-day neuroectodermal induction was performed with 10 μM SB-505124 hydrochloride hydrate (Sigma-Aldrich) and 10 μM IWP-2 (Merck Millipore), after which the EBs were plated down to 0.75 μg/cm^2^ laminin-521 (Biolamina) and 10 μg/cm^2^ human placental collagen Type IV (Sigma-Aldrich) in XF-Ko-SR medium. Pigmented foci were selected and replated, and hESC-RPE stocks were cryopreserved at passage 3. For transplantation, 250,000 hESC-RPE cells/cm^2^ were thawed on laminin-521 and collagen type IV-coated PET inserts containing 1 μm pores (Merck Millipore). The cells were cultured in XF-Ko-SR medium for 47 ± 8.6 days (mean ± SD) prior to live cell shipment and transplantation.

### Authentication of hESC-RPE

Human ESC-RPE authentication was performed as previously described [16]. Briefly, transepithelial electrical resistance (TEER) was triplicate measured with Millicell volt-ohm meter (Merck Millipore) [17]. The key RPE protein expression and localization were verified with indirect immunofluorescence labeling for zonula occludens-1 (ZO-1), claudin-3, claudin-19, sodium-potassium adenosine triphosphatase (Na^+^/K^+^-ATPase), bestrophin, and MER Proto-Oncogene, tyrosine Kinase (MERTK). Enzyme-linked immunoassay (ELISA) for pigment epithelium-derived factor (PEDF) was carried out from apical and basal media collected after overnight incubation and analyzed with the Human PEDF ELISA kit (BioVendor) following the manufacturer’s instructions. Phagocytosis assay was conducted with porcine photoreceptor outer segments (POS) by 4 h apical incubation at 37 °C in the presence of 10% fetal bovine serum (Thermo Fisher Scientific), followed by labeling with anti-rhodopsin antibody and tetramethylrhodamine (TRITC). The nuclei were counterstained with DAPI included in ProLong Gold mounting medium (Thermo Fisher Scientific). Images were acquired with an LSM 700-800 Confocal microscope (Carl Zeiss) and processed with the Zen 2.3 SP1 Black software (Carl Zeiss). All primary and secondary antibody details appear in Table S1 (Additional file 1).

### Cell shipment and viability testing

Temperature-controlled (+15 to +25 °C) live shipment of the hESC-RPE grafts was arranged via World Courier. The grafts were shipped as intact PET inserts placed in conical 50-mL tubes (Falcon Centrifuge Tubes, Corning) in Gibco Hibernate A medium (Thermo Fisher Scientific) supplemented with 15% KnockOut SR XenoFree CTS, 2 mM GlutaMAX, and 50 U/ml penicillin-streptomycin (all from Gibco, Thermo Fisher Scientific). The graft-containing tubes were placed in a thermally insulated box with heated (+37 °C) gel pads and temperature monitoring. RPE morphology, expression/polarization of RPE marker proteins, and TEER were monitored before shipment at the manufacturing site (Finland) and after arrival at the transplantation site (Singapore). Morphology was examined with phase contrast microscopy (Nikon Instruments Europe and Carl Zeiss Meditec), and TEER was measured with a Millicell volt-ohmmeter as described above. Identical measurement systems were used at both locations. At their destination, hESC-RPE inserts were placed in a cell culture incubator in XF-Ko-SR medium for at least 2 days of recovery prior to surgery. Immunostainings for claudin, Na^+^K^+^-ATPase, bestrophin, and MERTK were performed as described in the “Authentication of hESC-RPE” section (before shipment) and the “Immunolabeling of tissue sections” section (after shipment).

### Animals

Thirteen cynomolgus monkeys (*Macaca fascicularis*) (body weight 3.0–6.0 kg, 4–6 years old) were sourced from SingHealth Experimental Medicine Center, Singapore. All animal studies were approved by the Institutional Animal Care and Use Committee (IACUC) of SingHealth (Singapore). Bilateral surgery (*n* = 1) was only allowed in succession and if the previously operated eye was assumed to have regained good visual function (assessed by animal behavior and the absence of significant structural damage on multimodal imaging). In accordance with this IACUC regulation, animals scheduled for both eyes were planned for sham surgery in the first eye and submacular hESC-RPE implantation in the second (unoperated) eye. All animals were handled in accordance with the Association for Research in Vision and Ophthalmology (ARVO) statement for the use of animals in Ophthalmic and Vision Research and performed in American Animal Association LAC (AAALAC) International-approved facility.

### Human ESC-RPE graft transplantation

A total of 13 NHPs were utilized in this study. Eleven animals had hESC-RPE graft transplantation, and 2 animals underwent sham surgery alone (native RPE was removed but not followed by RPE graft implantation); one animal had bilateral surgery with a sham procedure in the right eye and hESC-RPE transplantation in the left eye, making a total of 14 eyes (Table 1). Immunosuppression, general anesthesia, and transplantation were performed as previously described [18]. In brief, a 25-G trans pars plana vitrectomy was performed using a Stellaris PC (Bausch & Lomb, Singapore) or Constellation (Alcon, Singapore) vitrectomy machine and a surgical microscope equipped with microscope-integrated intraoperative optical coherence tomography (miOCT, OPMI-Lumera 700, C. Zeiss Meditec, Singapore). The subretinal injection by a 38-G subretinal cannula (Cat #3247, MedOne Surgical Inc., Sarasota, FL, USA) was performed either by an automated injection system connected to the vitrectomy machine (Constellation, Alcon) or manually. Surgical removal of submacular RPE (ca. 2 × 3 mm) was achieved with a 20-G custom extensible loop instrument at elevated intraocular pressure (IOP) [19]. A custom-built, subretinal implant shooter instrument enabled subretinal implantation of the RPE graft (bullet-shaped, 1.1 × 2 mm) [20]. The remaining hESC-RPE material was immediately fixed with 10% formalin similarly serving as quality control for the shipment. Air-fluid exchange was then performed via active extrusion using a brushed silicone soft tip cannula (Cat #3222, MedOne Surgical Inc., Sarasota/ FL, USA), and included gentle subretinal fluid drainage from the bleb retinal detachment and retinotomy edge apposition.

**Table 1:** Summary of the technique details of 14 operated eyes of NHPs. Of the 13 animals, 12 had unilateral surgery in the right eye. Case numbers #8 and #14 refer to an animal that had bilateral surgery, with respective procedures performed 2 weeks apart. Thus, in total, 11 animals received a subfoveal hESC-RPE graft, and 3 NHPs had sham surgery/controls, without implantation of hESC-RPE

Case ID	Condition to create bleb (injection method/tamponade)	hESC-RPE graft under fovea	Ophthalmic follow-up	Histology/nuclei count	IHC	Surgical outcome group
#1	Manual injection/BSS	No/sham control	Yes	No, sample processing unsuccessful	No	N. A
#2	Manual injection/BSS	No/sham control	Yes	No, sample processing unsuccessful	No	N. A
#3	Automated injection/BSS	Yes	Yes	No, sample processing unsuccessful	No	Unfavorable
#4	Automated injection/BSS	Yes	No, died at 2 weeks	No, sample processing unsuccessful	No	N. A
#5	Automated injection/BSS	Yes	Yes	No, sample processing unsuccessful	No	Unfavorable
#6	Automated injection/BSS	Yes	Yes	Yes	Yes	Unfavorable
#7	Manual injection/BSS	Yes	Yes	No, foveal sections not obtained	Yes	Favorable
#8	Manual injection/air	Yes	Yes	Yes	Yes	Favorable
#9	Manual injection/air	Yes	Yes	Yes	Yes	Favorable
#10	Manual injection/PFCL	Yes	Yes	Yes	Yes	Favorable
#11	Manual injection/PFCL	Yes	Yes	Yes	Yes	Favorable
#12	Manual injection/PFCL (Ca^2+^, Mg^2+^-free BSS)	Yes	Yes	Yes	Yes	Unfavorable
#13	Manual injection/PFCL	Yes	Yes	Yes	Yes	Unfavorable (transition case)
#14	Manual injection/air	No/sham control	Yes, euthanized at 6 weeks	No, sample processing unsuccessful	No	N. A.

### In vivo animal follow-up

Postoperative transplantation site follow-up was monitored non-invasively by spectral domain OCT (SD-OCT) (Spectralis®, Heidelberg Engineering, Heidelberg, Germany). Imaging of the retina via a confocal scanning laser ophthalmoscope (blue fundus autofluorescence/BAF, infrared reflectance/IR, and fluorescein angiography/FA) was achieved using the same device. All animals had the above ophthalmic imaging at baseline and postoperative days 5, 14, and 28. SD-OCT images were assessed by two masked independent graders (summarized in Table S2, Additional file 1).

Retinal function was assessed by full-field electroretinography (ERG) using an Espion system (Diagnosis LLC, USA) prior to and 28 days post-surgery using protocols based on those recommended for human patients by the International Society for Clinical Electrophysiology of Vision (ISCEV) [21], but with a light-adapted (LA) flash strength of 5.0 cd.s.m^−2^ (LA 5.0) [22, 23], Animals were anesthetized and the pupils dilated, followed by 20 min dark-adaptation prior to full-field ERG recording.

### Histopathological processing

After 28 days, the animals were sacrificed in deep intramuscular anesthesia with an intracardiac injection of the euthanizing agent (Phenobarbital, VALABARB®, Jurox Pty Limited), followed by perfusion fixation via carotid artery with 4% formaldehyde or Davidson’s fixative medium (13% formaldehyde, 15% ethanol, 5% glacial acetic acid) [24]. Six of 13 animals had both eyes enucleated; the remaining animals had only the operated eye removed. The entire globes were immersed in the same fixative medium overnight. This resulted in 11 transplanted eyes, 3 surgical (sham) control eyes, and 5 naive eyes being processed for histological analysis. Following removal of the anterior segments, full-thickness samples (3 × 2 mm, retina→sclera) were cut and embedded in paraffin (standard histological processing). The sections were serially cut at 5 μm thickness with a microtome (Leica RM2255) and stained.

### Histology

The paraffin sections were stained for the evaluation of retinal integrity with hematoxylin and eosin (H&E) using standard protocols and imaged with Hamamatsu NanoZoomer S60 WSI scanner (Hamamatsu Photonics). H&E-stained sections containing macula were imaged for quantification of the outer nuclear layer (ONL) nuclei number with a Nikon Eclipse TE2000-S phase contrast microscope (Nikon Instruments Europe B.V.) using ×10 objective and the nuclei counted from 3–9 sections per animal. Three corresponding measurement sites of 100 μm in length were chosen from each section and the nuclei manually counted with the ImageJ cell counter plugin [25].

### Immunolabeling of tissue sections

Samples were deparaffinated using standard protocols. Heat-induced epitope retrieval was performed either with 10 mM sodium citrate, 0.05% Tween-20, pH 6, or 10 mM Tris (Table S1, Additional file 1) in a preboiled buffer for 30 min at room temperature (RT). After blocking with 10% normal donkey serum (Merck Millipore) and 5% bovine serum albumin (Sigma-Aldrich) for 1 h at 37°C, the samples were incubated overnight with primary antibodies at 4°C, and secondary antibodies for 1 h at RT (Table S1, Additional file 1). Prior to mounting with ProLong Gold containing DAPI (Thermo Fisher Scientific), lipofuscin autofluorescence was quenched with TrueBlack (Biotium) according to the manufacturer’s instructions. The sections not incubated with primary antibodies acted as controls. Images were taken using the Olympus IX51 fluorescence microscope (Olympus).

### Statistical analysis

Data are presented as mean ± SD. Comparisons of non-parametrically distributed data were performed using the two-tailed Mann-Whitney U test with the GraphPad Prism 5 Software (La Jolla, CA, USA, www.graphpad.com). Differences were considered significant at *P < 0.05, **P < 0.01, and ***P < 0.001.

## Results

### Long-distance shipment of live hESC-RPE monolayers

**Fig. 1:**
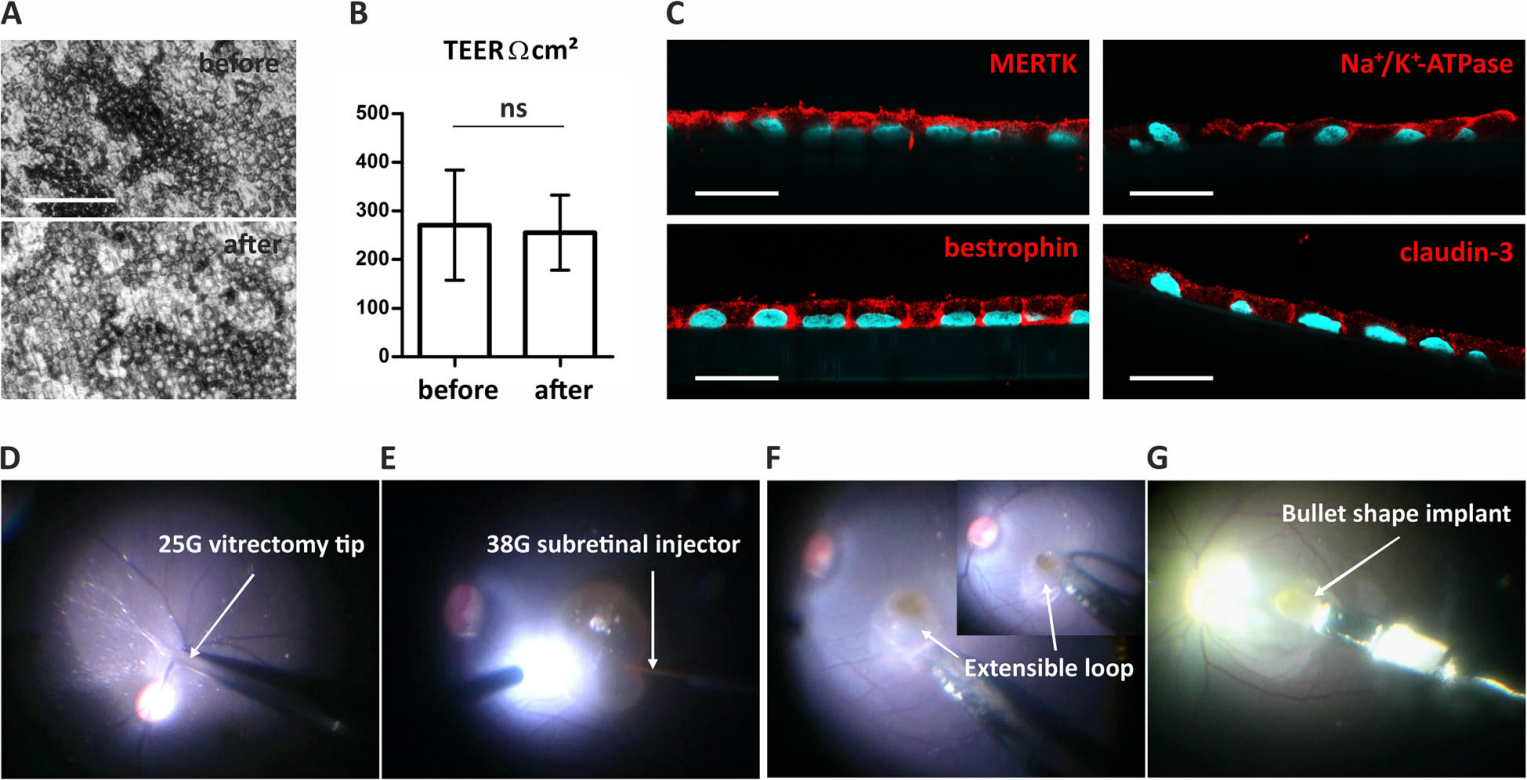
Delivery of hESC-RPE graft from the production site to the subretinal space. **A** Phase contrast micrographs of RPE morphology before and after shipment. **B** TEER of hESC-RPE before and after shipment (mean and SD, from 5 independent shipments, 2–6 inserts per shipment). *P* > .05 (not significant, ns, Mann-Whitney U test). **C** Confocal micrographs of hESC-RPE grafts labeled post-shipment with antibodies against proteins important for RPE functionality (shown for the 54 h shipment). Scale bars, **A** 100 μm and **C** 20 μm. **D**–**G** Key surgical steps in submacular implantation of hESC-RPE grafts. **D** Triamcinolone-aided detachment of posterior cortical vitreous from the retinal surface. **E** Subretinal BSS injection to induce detachment of the macula. **F** Surgical removal of naive RPE with a custom-made extensible loop instrument (the overview is shown in the insert image). **G** Submacular implantation of hESC-RPE grafts on a porous polyester scaffold using a custom-made instrument

The in vitro RPE characteristics and functionality were authenticated by transepithelial electrical resistance (TEER, 130–430 Ω*cm^2^), polarized expression of RPE markers (claudin-19, ZO-1, Na^+^K^+^-ATPase, bestrophin, MERTK), phagocytosis of porcine POS, and apically polarized PEDF secretion (Fig. S1, Additional file 1). The live hESC-RPE monolayers were manufactured at the cell production facility in Finland and shipped for on average 33 h (5 separate shipments, Fig. S2, Additional file 1) at RT (range from 20.6 to 28 °C) to the surgical site in Singapore using a high glucose CO_2_-independent medium. Once, due to customs clearance delay, the total shipment duration was 54 h. After shipping, even in the delayed 54-h transport batch, all monolayers maintained viability and expression of RPE markers (Fig. 1) as indicated by morphology, TEER (170–360 Ω*cm^2^), and immunocytochemistry (claudin-3, Na^+^K^+^-ATPase, bestrophin, MERTK).

### Submacular transplantation of hESC-RPE grafts and postoperative in vivo follow-up

**Fig. 2:**
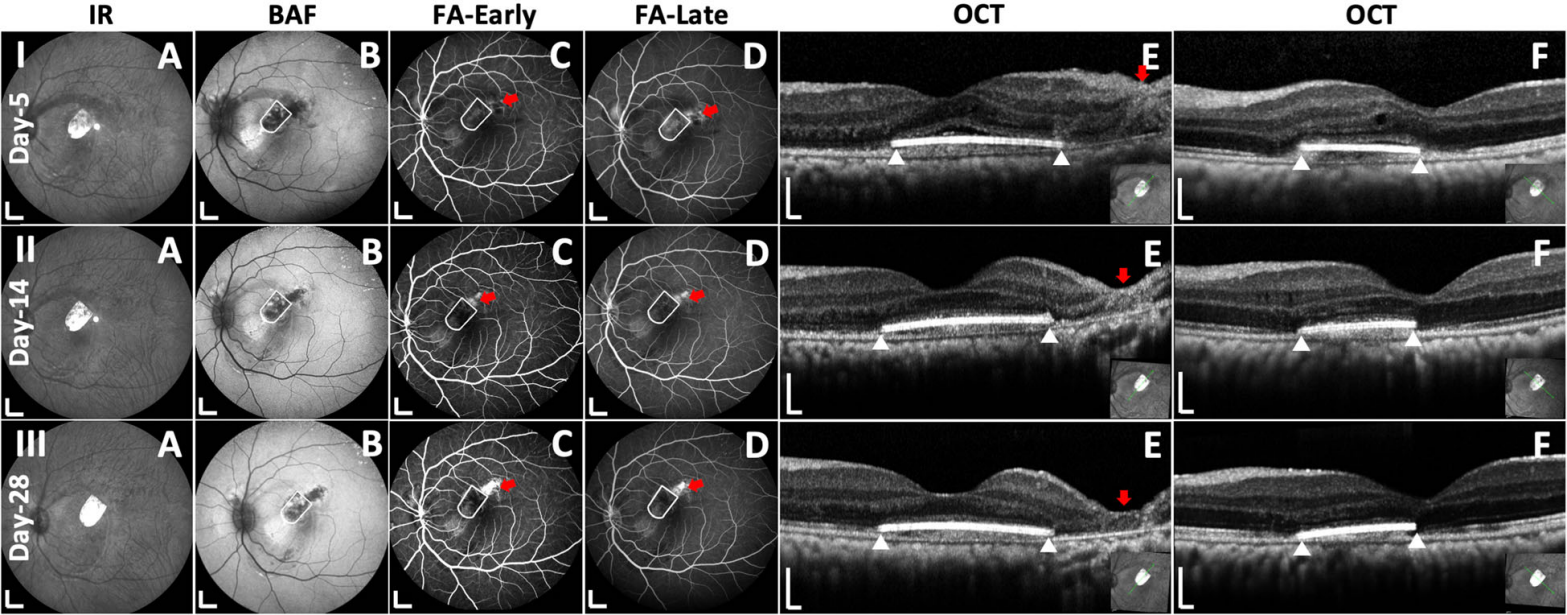
Postoperative in vivo analysis with multimodal imaging. Lines I, II, and III: representative case of submacular hESC-RPE graft at 5, 14, and 28 days after surgery. **A** Infrared reflectance (IR) images of the posterior fundus. **B** Blue laser autofluorescence (BAF) images show minimal hyper autofluorescence changes overlapping the hESC-RPE graft which correlate with denude native RPE procedure. **C**, **D** Early- and late-phase fluorescein angiography (FA) show no fluorescein leakage over or immediately surrounding the hESC-RPE graft (white outlines); some staining is present at the site of the denude RPE wound and surrounding the retinotomy (solid red arrows). **E**, **F** Horizontal and vertical SD-OCT scans through the middle of the implants (as shown in inserts). Outer retinal reflectance layers on SD-OCT are grossly preserved both over the hESC-RPE graft (white triangles indicate the edges of the graft) and the remaining bleb area, except the retinotomy site (red arrows). Scale bars, **A**–**D** 2mm and **E**, **F** 200 μm


Key steps of submacular transplantation of hESC-RPE graft were video recorded for evaluation (Fig. 1 and Additional file 2: Video S1). A total of 14 submacular procedures were performed on 14 eyes of 13 animals (hESC-RPE graft implantation *n* = 11, bleb retinal detachment (bRD) *n* = 3). The operated eye generally recovered in a few days; during ophthalmic imaging follow-up, the conjunctiva, cornea, lens, and vitreous showed no inflammation and the hESC-RPE grafts maintained a stable submacular position with the retinotomy sealed (Fig. 2 and Additional file 1: Table S2).

**Additional file 2: Video S1.** Key steps of submacular transplantation of hESC-RPE graft in a surgical removal naïve RPE model in non-human primates. 1. Create posterior vitreous detachment. 2. Detach fovea guided by miOCT. 3. Perform retinotomy. 4. Scrape naïve RPE. 5. Transplant hESC-RPE/ PET graft. 6. Adjust position guided by miOCT. 7. Drain subretinal fluid under miOCT.

**Fig. 3:**
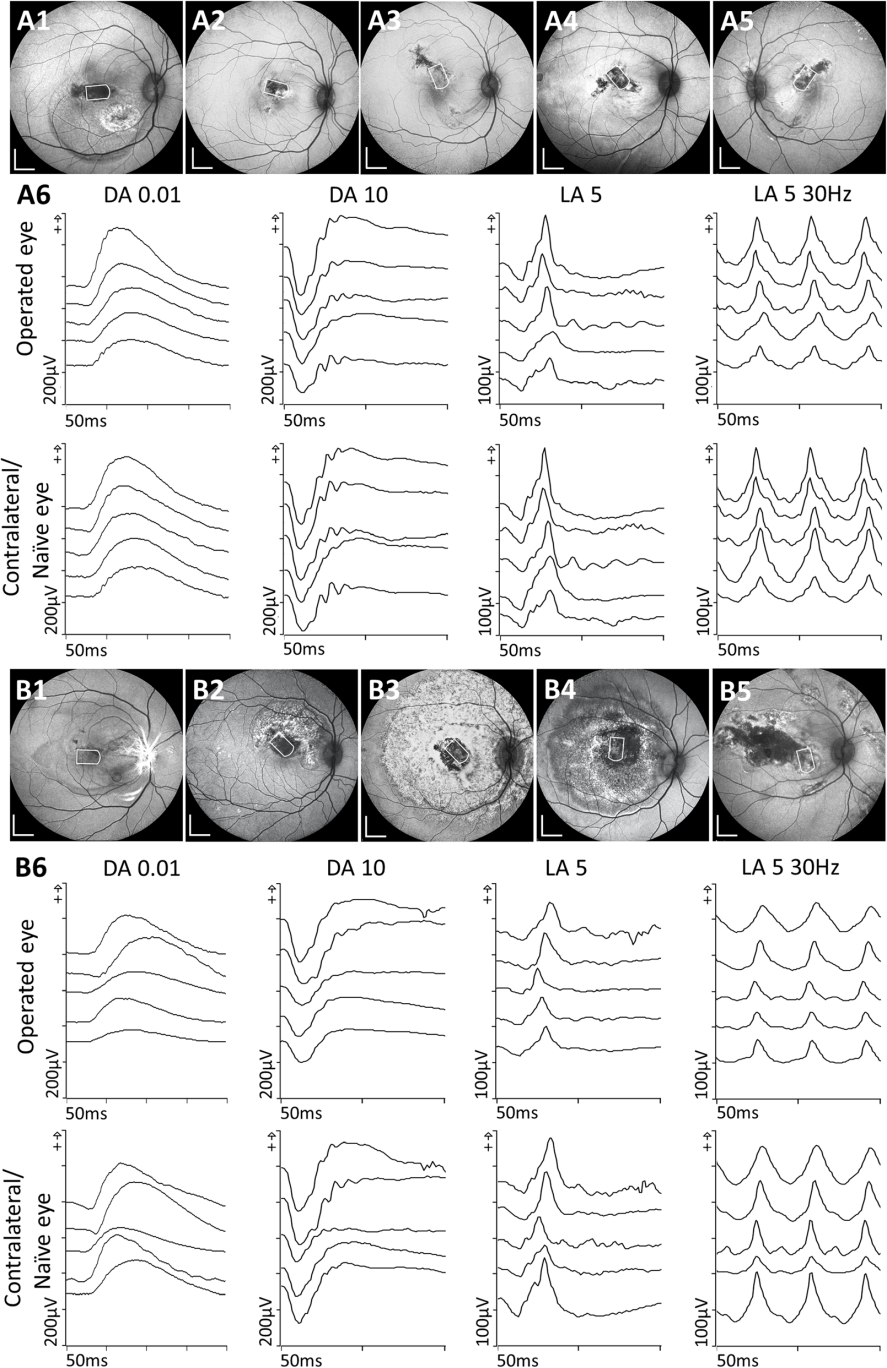
Comparison of surgical outcome groups by ophthalmoscopy and electroretinography. **A1**–**A5**, **B1**–**B5** BAF images of ten eyes with hESC-RPE grafts were divided into two groups, favorable surgical outcome (**A1**–**A5**) and unfavorable surgical outcome (**B1**–**B5**), one animal was lost to follow up. Scale bars, 2 mm. **A6**, **B6** All five cases of full-field ERG assessment from each group in scotopic (dark-adapted, DA 0.01), mixed (DA 10), photopic flash (light-adapted, LA 5), and flicker (LA 5 30 Hz), with a contralateral (naive) eye at day 28 after surgery. The subjects, from top to bottom, represent the cases from **A1** to **A5**, **B1** to **B5**, respectively

The surgical outcomes of 10 hESC-RPE/PET implantation surgeries (case #4 died on day 14 post-surgery) were divided into 2 groups (favorable versus unfavorable surgical outcome groups) according to SD-OCT, BAF and, histology at day 28 (Fig. 3, Table 1 and Additional file 1: Table S2). A key factor to surgical success relates to bRD creation [26].

In those with a favorable surgical outcomes, the bRD was created gently in well-controlled conditions (controlled balanced salt solution (BSS) injection speed and volume, reduced IOP under either full air tamponade or partial perfluorocarbon liquid (PFCL) tamponade during subretinal injection, Table 1) to minimize tangential stretching forces on the fovea [26]. A more preserved foveal microstructure was present on miOCT when compared to the unfavorable outcome group (data not shown). In the postoperative in vivo follow-up, intact foveal reflectance layering on postoperative SD-OCT, decreased or minimal BAF, and FA changes occurred within the original bRD (Fig. 2). The region of surgically removed RPE consistently yielded altered signals on multimodal imaging throughout follow-up. The retinal layers above the hESC-RPE graft were maintained with a continuous external limiting membrane (ELM) (Fig. 2). There was minimal subretinal fluid observed above or below the grafts at day 5, fully absorbed by day 28 (Fig. 2 and Additional file 1: Table S2). Further, 4 of 5 cases showed no interocular asymmetry in full-field ERG, with one case showing mild interocular asymmetry (Fig. 3A6). The latter animal (Fig. 3A4) experienced respiratory and cardiac arrest on induction of general anesthesia prior to implantation surgery but was successfully resuscitated and subsequently performed without abnormalities.

Several cases had an unfavorable surgical outcome. These were related to significant foveal structural damage when the bRD was created with an automated injection system, an intraoperative IOP spike, or when Ca^2+^ and Mg^2+^-free BSS was used. Postoperative widespread BAF and FA changes were observed progressing beyond the original bRD area (Fig. 3B and Additional file 1: Fig. S3). On SD-OCT, an additional layer was observed above the graft at day 5, progressively resulting in partially discontinuous ELM at day 28. The surrounding native RPE even beyond the retinal bleb was seen as interrupted on BAF and OCT (Fig. S3 and Table S2, Additional file 1). Detaching the retina in case #12 under Ca^2+^ and Mg^2+^-free BSS also resulted in foveal trauma along with fast progressing intraoperative lens opacification/cataract during the operation [27, 28]. Retinal gliosis (visualized by immunostaining for glial fibrillary acidic protein (GFAP)) was strongly upregulated, indicating retinal stress (Fig. S5, Additional file 1). Full-field ERG in the unfavorable surgical outcome group were overall satisfactory (Fig. 3B6). One case had clear generalized ERG attenuation (case ID #12, Fig. 3B5), but none of the other four cases showed significant changes in ERG. It should however be noted that damage confined to the fovea would not be expected to give a full-field ERG abnormality.

### Photoreceptor preservation and cellular stress in the hESC-RPE transplanted macula

**Fig. 4:**
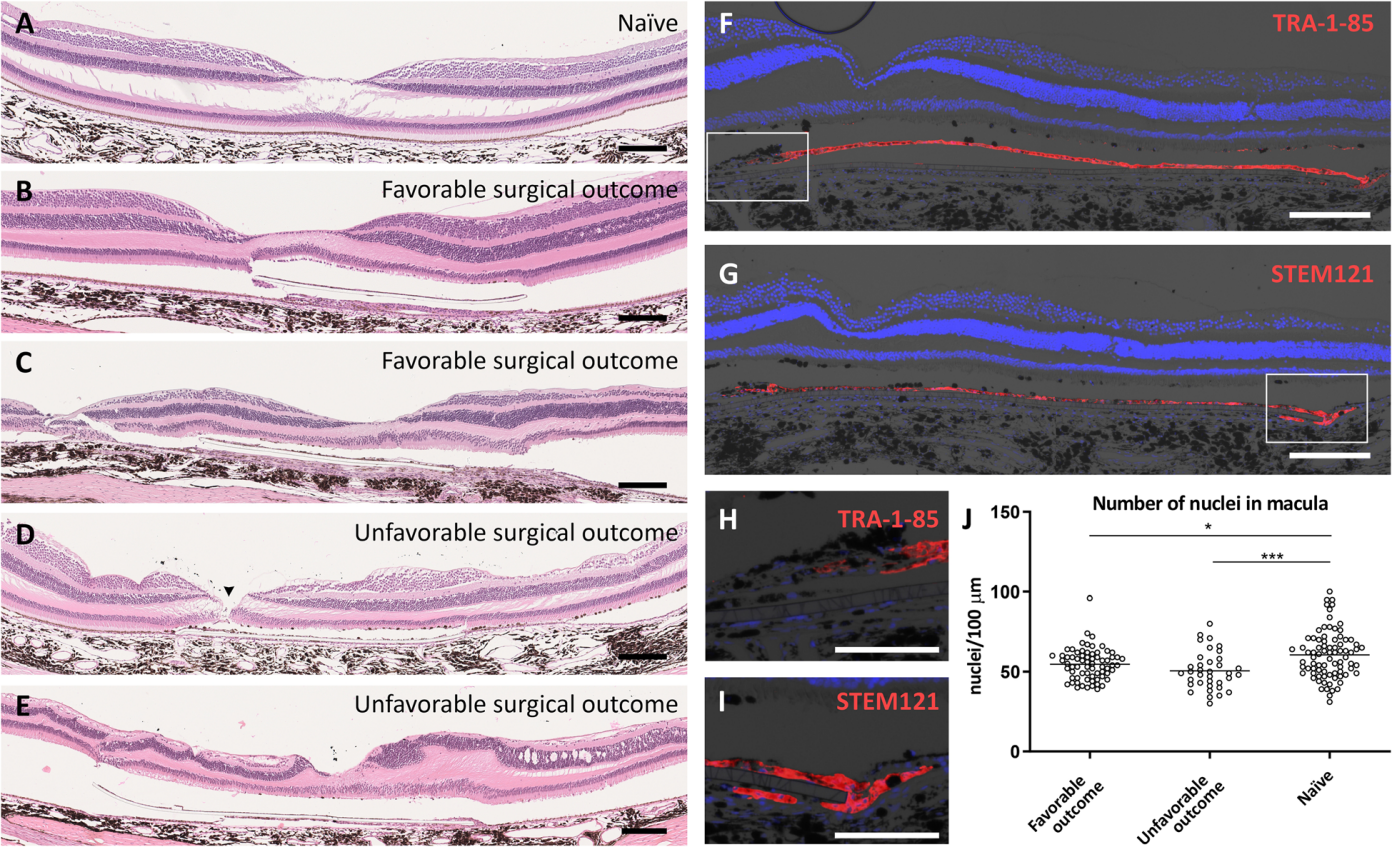
Histological and immunohistochemical evaluation of the graft and retina integrity. **A** H&E stains of the normal retina in naive eyes (*n*=5). **B**,**C** H&E stains show good retinal preservation in animals from the favorable surgical outcome group (*n* = 4, cases 8 and 11 shown, respectively). **D**, **E** Histology reveals a more disrupted retinal morphology in the animals from the unfavorable surgical outcome group (*n* = 3, cases 6 and 13 shown, respectively). The arrowhead in **D** indicates a foveal microtear. **F**–**I** Epifluorescent and brightfield micrograph overlays of human-specific TRA-1-85 and STEM121 immunolabelings show human cells on the PET carrier (case 9 shown), migration of human cells away from the carrier (**H**, higher magnification from **F** marked by white box), and the presence of non-human cells on the carrier (**I**, higher magnification from **G** marked by white box). The nuclei were counterstained with DAPI. Scale bars, **A**–**G** 200 μm and **H**, **I** 100 μm. **J** Quantification of the nuclei number over the graft. The nuclei were counted manually from 3 to 9 H&E-stained macular sections per animal (3 corresponding measurement points 100 μm in length). The scatter dot plot shows a slightly (*P* < 0.05*, Mann-Whitney U) decreased number of nuclei (55 ± 10; mean ± SD) in the maculae above the graft in the favorable surgical outcome group compared to naive eyes (61 ± 15). The reduction in the number of nuclei (51 ± 12) was somewhat greater (*P* < 0.001***) in the animals from the unfavorable surgical outcome group compared to naive eyes

Due to technical issues, histological analysis was successful in 8 of 11 eyes that had received a hESC-RPE graft, 7 of which had foveal sections recovered (Table 1). Histology confirmed that the hESC-RPE xenografts were maintained under the NHP host retina (Fig. 4B–E). A layer of pigmented cells was observed on the PET carrier in all analyzed eyes (*n* = 8) and verified as human origin by immunostaining for human-specific antigens, TRA-1-85 and STEM121. Some human marker-positive cells were observed under or adjacent to the hESC-RPE graft in 5 of 8 eyes analyzed with immunostaining, and some negative cells on the PET carrier at its edges, suggesting integration of human cells away from the graft and primate cells onto the carrier (Fig. 4F–I). Overall, 6 of 8 eyes showed well-preserved retinal structure (Fig. 4A–D) with slightly (*P* < 0.05) less nuclei in the macula above the hESC-RPE graft in the animals with favorable surgical outcome (*n* = 5) compared to unoperated naive control (*n* = 5, Fig. 4J). The decrease in the number of nuclei was greater in the animals with unfavorable surgical outcomes (*n* = 5; *P* < 0.001) compared to naive control (Fig. 4J), and in 2 cases, the overall retinal morphology was more severely disrupted (Fig. 4E). In some tissue sections, the foveal microtear observed during surgery was detected (Fig. 4D).

**Fig. 5:**
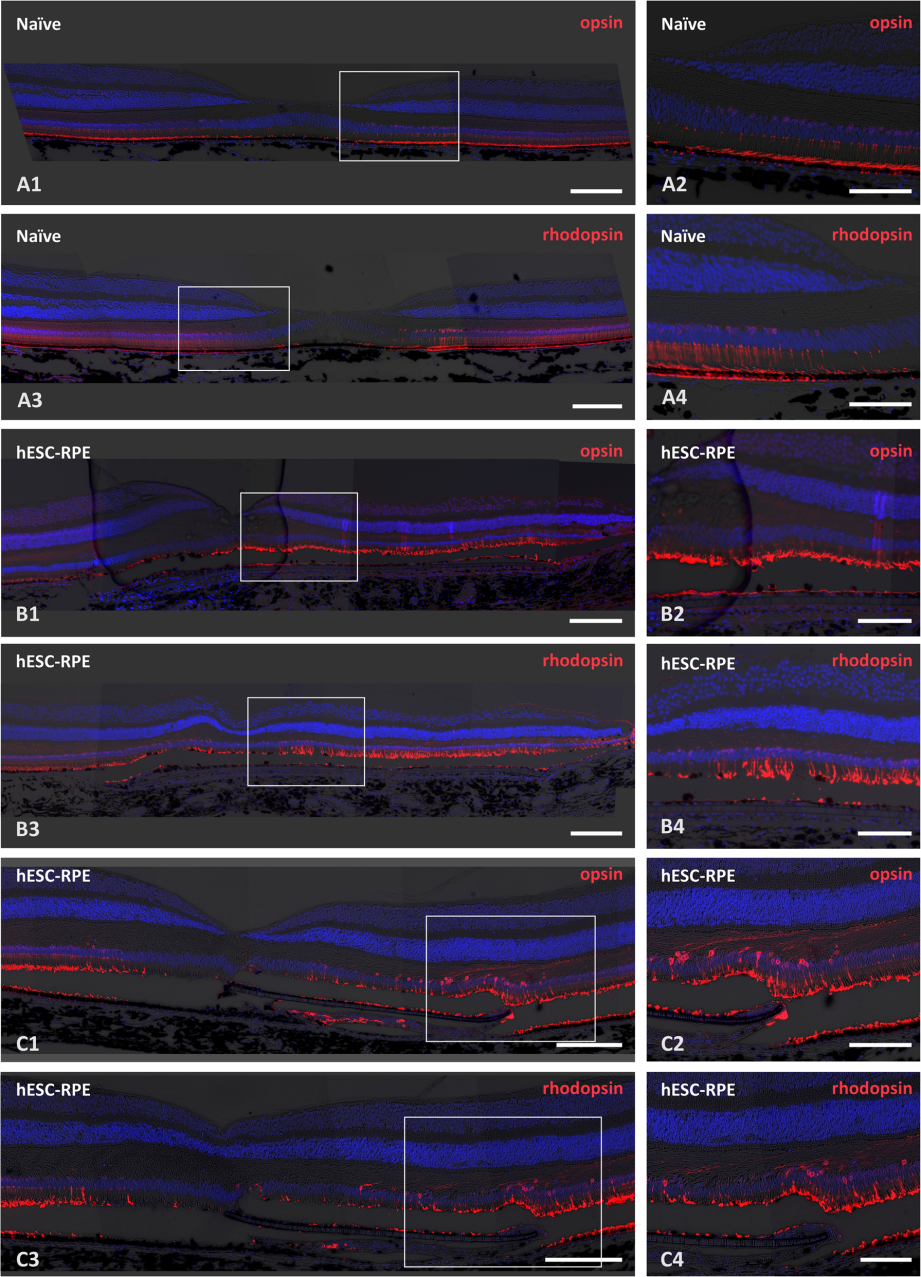
Evaluation of photoreceptor preservation. Epifluorescent and brightfield micrograph overlays of L/M-cone opsin and rhodopsin immunolabelings show varying degrees of photoreceptor outer segment (POS) preservation above the graft. **A1**–**A4** Staining at the fovea from naive control animal. **B1–B4** and **C1**–**C4** Staining at the fovea from 2 different hESC-RPE-transplanted animals (cases 9 and 8, respectively). White boxes mark the sites for enlargements to the right column. Although positive signals of both opsin/rhodopsin and areas of POS preservation (**B1**–**B4**) are present in all examined eyes (*n*=8) above/around the hESC-RPE graft, the eyes also contain areas where increased localization of opsins to the photoreceptor cell bodies is present or the POS are completely disrupted (**C1**–**C4**). Scale bars, **A1**, **A3**, **B1**, **B3**, **C1**, **C3** 200 μm and **A2**, **A4**, **B2**, **B4**, **C2**, **C4** 100 μm. The nuclei counterstained with DAPI

Photoreceptor preservation was further assessed using immunostaining for L/M-cone opsins and rhodopsin. There were varying degrees of POS preservation above the hESC-RPE graft. A positive signal for opsin/rhodopsin was observed in all eyes (*n* = 8, Fig. 5). However, all eyes also contained areas above/around the hESC-RPE graft where increased opsin localization to the photoreceptor cell bodies was observed or the POS were completely disrupted (Fig. 5C).

**Fig. 6:**
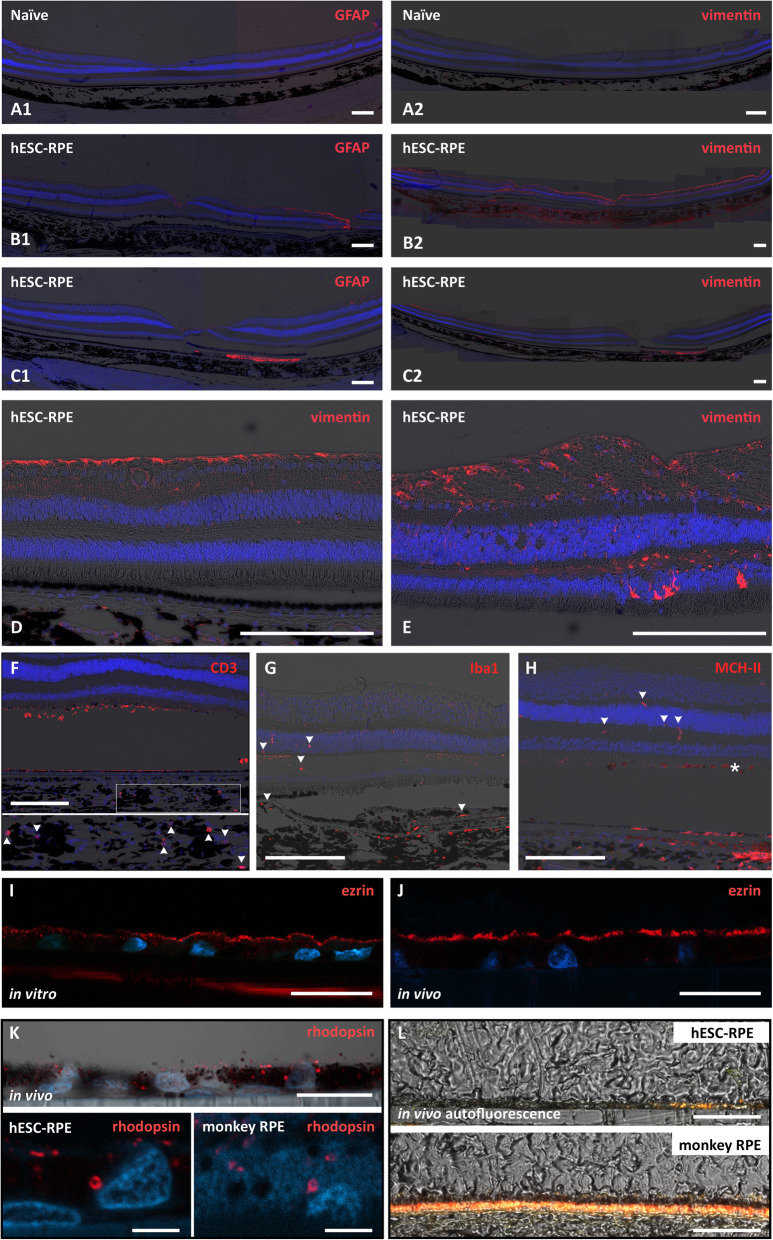
Evaluation of retinal gliosis and in vivo functionality of the hESC-RPE cells. **A1**, **B1**, **C1** Epifluorescent and brightfield micrograph overlays show only minor retinal GFAP upregulation, mainly around the retinotomy (**B1**) and in 4 of 6 cases also slightly in the fovea (**B1**, **C1**). **A2**, **B2**, **C2** Vimentin is more upregulated with higher variation between animals than GFAP. **A1**, **A2**, **B1**, **B2**, **C1**, **C2** Stains from 3 different animals. Both GFAP and vimentin are also upregulated in some areas under the graft (**C1**, **C2**). **D**, **E** Vimentin upregulation in ONL is observed in 2 of 8 animals. **F**–**H** Evaluation of the effectiveness of immunosuppression. **F** Epifluorescent and brightfield micrograph overlays show a few CD3-positive cells (arrowheads in the enlarged image indicated by the white box). The subretinal signal originates from POS. **G** Some amoeboid/activated Iba1-positive cells are present in the inner retina and subretinal space (arrowheads) but most reside in the choroid. **H** MHC-II positivity is detected occasionally in the retina (arrowheads), in pigmented cells in the subretinal space (asterisk), and in a few cells on the PET scaffold at the graft edge and in the choroid. **I**, **J** Confocal micrographs of ezrin immunolabeling identify microvilli at the apical surface of the hESC-RPE both prior to transplantations in vitro and after transplantation in vivo. **K** Confocal micrographs of rhodopsin immunolabeling show localization of POS inside the hESC-RPE cells compared with the monkey RPE. The nuclei counterstained with DAPI. **L** Confocal micrographs of unstained paraffin sections with excitation 488/568/647 nm show autofluorescence in some areas on the PET carrier in addition to monkey RPE. The image shows a central area of the hESC-RPE graft. Scale bars, **A**–**H** 200 μm, **I**–**K** (upper image) 20 μm, **K** (lower images) 5 μm, and **L** 50 μm

Glial cells were visualized by immunolabeling with antibodies against GFAP and vimentin. GFAP was mainly upregulated in relation to the retinotomy and often also slightly in the fovea (*n* = 4 of 7 eyes with foveal sections recovered), likely due to surgery-related scarring (Fig. 6 B1, C1). When Ca^2+^ and Mg^2+^-free BSS were used as the tamponade agent during surgery, a strong GFAP upregulation was observed throughout the retina (Fig. S4, Additional file 1). Vimentin was more upregulated than GFAP, and there was more variation in the level of upregulation between animals (Fig. 6A–C). However, the upregulation was mainly confined to the retinal layers other than ONL, in which vimentin immunoreactivity was observed in only 2 animals (Fig. 6D, E). Upregulation of either vimentin, GFAP, or both was present in all animals (*n* = 8) under/next to the hESC-RPE graft (Fig. 6 C1, C2).

### Effectiveness of sirolimus immunosuppression on retinal T cell infiltration and microglia activation

The efficacy of the immunosuppression regime was studied as graft rejection is probable with a xenograft and is a potential risk in allograft transplantations. Histologically, the eyes were analyzed for retinal T cell infiltrations (anti-CD3), activation of microglia (anti-Iba1), and expression of major histocompatibility complex class II (MHC-II) by antigen-presenting cells and hESC-RPE as interferon gamma (IFN-γ), an inflammatory cytokine, upregulates MHC-II expression of hPSC-RPE [29, 30]. In all analyzed eyes (*n* = 8), only occasional CD3-positive cells were observed mainly in the choroid indicating successful T cell suppression (Fig. 6F). Retinal microglia activation was also efficiently suppressed. Minor migration of the microglia was detected from the inner retina and the presence of only a few Iba1-positive cells with amoeboid/activated morphology in the outer retina and subretinal space (*n* = 8, Fig. 6G). MHC-II-expressing cells were present in a similar manner as Iba1, with a few MHC-II-positive cells in the retina and subretinal space (*n* = 8). Apart from occasional cells among the cell monolayer on the PET carrier towards the edge of the graft, the hESC-RPE cells were negative for MHC-II (Fig. 6H). Some Iba1 and MHC-II-positive cells were also observed in the choroid (*n* = 8, Fig. 6G, H). No major differences in choroidal staining of CD3/Iba1/MHC-II between the hESC-RPE transplanted eyes and the non-operated collateral eyes were found (*n* = 3 animals).

### Functional integration of the hESC-RPE graft in the macula of a surgical RPE injury NHP model

The health of individual hESC-RPE grafts before and after transplantation was traced by staining the spare hESC-RPE on PET carrier leftover from surgery (*n* = 3) with anti-ezrin, a microvilli marker, and comparing that to the ezrin staining post-surgery. Both in vitro and in vivo, equal subcellular localization of ezrin was observed (Fig. 6I, J). The functionality of the hESC-RPE graft in vivo was evaluated by the cells’ ability to phagocytose POS. Immunohistochemical analysis showed opsin/rhodopsin-positive particles inside the hESC-RPE cells in 8 of 8 eyes (Fig. 6K). Further evidence for in vivo phagocytosis by the transplanted cells was obtained from autofluorescent signal observed in some areas of all analyzed grafts (*n* = 8), indicating the presence of POS-derived lipofuscin in the hESC-RPE (Fig. 6L).

## Discussion

The development of a safe and effective cell replacement therapy requires evaluation of several aspects in preclinical animal studies. This study uses a novel NHP model, with a disrupted ocular immune privilege, to address logistical, surgical, and immunosuppression-related issues. The study highlights the advantages of a foveate animal model (nearly identical to the human retina), as novel surgical macular dynamics were observed during RPE transplantation. Short-term submacular integration of a cell carrier-supported hESC-RPE monolayer that was shipped intercontinentally prior to transplantation was observed without obvious signs of rejection.

Differentiation of RPE cells from hPSCs is time-consuming, requiring specialized equipment and technical knowledge. In combination with good manufacturing practice (GMP)-compliant infrastructure and hPSC banking, centralized cell production is a cost-efficient guarantee for the quality, safety, and validity of the cells. Most hospitals cannot meet these requirements. Thus, shipment to clinical centers is needed from the production site. For an RPE suspension transplantation approach, the cells can be shipped in cryopreservation, but transplantation of RPE sheets may require graft shipment as live cultures. Currently, one clinical study has reported the shipment time to be 8 h [6], presumably at RT. The longest evaluation of hPSC-RPE under shipping conditions was 48-h stability at 4 °C [31]. Optimal storage conditions for cultured RPE were repeatedly reported at 16 °C [32, 33]. In this study, intercontinental shipment (from Finland to Singapore) of the hESC-RPE grafts proceeded by car and airplane at 21–28°C, exposing the cells to lengthy shipment time and potentially high mechanical stress. Using a high glucose CO_2_-independent transport medium, the TEER and polarized protein distribution critical for RPE functionality remained comparable to that pre-shipment even up to a 54-h delivery time.

The preferential disease manifestation of AMD in the macula is curious, given its multifactorial risk associations [34]. The lack of AMD animal models significantly challenges the development of novel therapeutics. Lesion models resulting in RPE and outer retinal loss have been utilized in rabbits, pigs, and NHPs to simulate the advanced atrophic form of AMD [35]. The surgical removal of the submacular RPE in the present study resulted in an immediate and localized outer retinal atrophy consistent with results in a rabbit model, using similar surgical techniques, with up to 3 months of follow-up [36].

MiOCT enables real-time monitoring with significant clinical utility for submacular procedures involving cell and gene therapy [37–39]. With an automated-foot pedal-controlled bRD technique, fluid egress was observed from the foveal center due to a tear evident on miOCT [26]. Early human RPE65 gene therapy trials had reported up to 20% foveal vulnerability [40]. The increased BAF signals at the bleb site and noted foveal trauma prompted a reconsideration of the bRD surgical technique. A further technical discussion of submacular fluid injection for foveal detachment appears elsewhere [26]. The ERG data failed to indicate any widespread retinal damage in the operated eyes, suggesting that any damage leading to an unfavorable outcome resulted from localized foveal damage relating to the operative procedure rather than any generalized effect triggered by the transplant, and thus raises no major safety concerns. It will be important for future studies, given the apparent anatomical success, to demonstrate and quantify functional integration objectively with multifocal ERG.

Disruption of the RPE/photoreceptor complex can trigger deleterious reactions within the entire neuroretina [41], reflected in clinical practice by the visual outcomes following macula-off rhegmatogenous retinal detachments (RRD); patients with longer-lasting subretinal fluid from unrepaired RRD are less likely to recover pre-operative vision [42]. In contrast, patients with acute central serous chorioretinopathy and submacular fluid can recover full visual acuity along with (near) normal retinal structure both on SD-OCT or BAF [43].

Following submacular placement of the hESC-RPE/PET graft, miOCT revealed trapped residual subretinal fluid after fluid-air exchange. Vigorous subretinal fluid drainage was initially avoided to avoid transplant slippage and iatrogenic damage at the retinotomy site. However, there were persistent residual subretinal fluid pockets revealed by early postoperative SD-OCT in cases with reduced injection volume bRDs followed by the appearance of outer retinal hyperreflective dots. It was hypothesized that maximal flattening of the retina around the implant might reduce the appearance of hyperreflective dots, contributing to decreased postoperative inflammation in the subretinal space. This was achieved using a brushed tip silicone cannula under miOCT (Video S1, Additional file 2) and implemented in future cases.

Histological assessment of retinal integrity demonstrated that transplanted hESC-RPE remained as a layer of pigmented cells on the PET carrier in all eyes; some human marker-positive cells were also integrating away from the graft. A recent clinical trial reported a similar outcome with hESC-RPE cell migration off the patch in 2 patients treated over the first 6 months after surgery before stabilization [6]. Importantly, in the present study, the retinal structure was well preserved in 6 of 8 eyes with only a slightly decreased number of nuclei in the macula above the hESC-RPE graft in the 5 animals with favorable surgical outcome. In addition, immunostaining for L/M-cone opsins and rhodopsin showed photoreceptor/outer segment preservation, although to a variable degree, above the hESC-RPE graft in all eyes.

Retinal stress was assessed by staining for intermediate filament proteins GFAP and vimentin, which are typically upregulated in retinal glia cells in response to disease or injury [44]. Remarkably, GFAP, considered a hallmark of Müller gliosis, was only slightly upregulated in the retinotomy site and fovea, consistent with the observed foveal vulnerability to trauma. In contrast, using a BSS without Ca^2+^/Mg^2+^ infusion [28] resulted in massive GFAP upregulation and reduced ERG amplitudes at 4 weeks (Fig. 3B5 and Additional file 1: Fig. S5). Furthermore, the predominance of vimentin immunoreactivity over GFAP also suggests alleviation of the retinal detachment-induced upregulation of intermediate filaments in retinal glial cells [45], only in 2 eyes was vimentin immunolabeling observed extending to the ONL, indicating a more prominent glial response. GFAP/vimentin immunoreactivity was also observed under or around the graft. Vimentin is constitutively expressed in monkey RPE [46]. As mechanical native RPE removal leaves some residual, likely damaged RPE cells at the graft site, the observed GFAP/vimentin immunolabeling probably originates in those cells.

Multiple studies have demonstrated that xenogeneic/allogeneic hPSC-RPE cells transplanted into non-immunosuppressed subretinal space are rejected by the immune system [12, 47, 48]. In addition to the loss of the graft by rejection, there may be a contribution from increased subretinal inflammation directly related to the procedure. This immunogenicity, however, also depends critically upon the local immune environment of the transplant site and the presence of antigen-presenting cells. Thus, the outer blood-retina barrier was intentionally disrupted at the graft site, a potentially strong xenogeneic immune response anticipated, and systemic immunosuppression used. The histological assessment confirmed the adequacy of the regime to suppress retinal T cell infiltrations and activation of microglia, despite the expression of MHC-II by hESC-RPE. Upregulation of the latter by inflammatory cytokine IFNγ in vitro has previously been shown [29].

Importantly, the hESC-RPE cells showed in vivo function as demonstrated by primate POS phagocytosis and lipofuscin accumulation. The POS particles contain 11 cis-retinal and all-trans-retinal which form lipofuscin precursor fluorophores and end in RPE lysosomes during phagocytosis. Although abundant accumulation of lipofuscin to the RPE is linked to retinal disorders, lipofuscin in the hESC-RPE also indicates that critical RPE functions are occurring, as lipofuscin fluorophore formation depends upon POS phagocytosis and functional visual cycle [49].

## Conclusions

The results show structural and functional submacular integration of hESC-RPE xenografts in an immunosuppressed non-human primate model with a compromised outer blood-retinal barrier. The shipping protocol used allowed effective intercontinental delivery of live cell grafts, demonstrating that distant cell production and intercontinental distribution of living cell product are a viable proposition for RPE cell transplant applications. It is anticipated that further refinement of the surgical techniques will improve the utility of macular cell engraftment in a therapeutic setting.

## Supplementary Information

**Additional file 1: Figure S1.** *In vitro* characterization of the hESC-RPE cells. **Figure S2.** Graphical representation of shipment temperatures and times. **Figure S3.** Postoperative *in vivo* analysis with multimodal imaging in an animal with unfavorable outcome. **Figure S4.** Effect of different subretinal BSS injection modes on fluorescein angiography. **Figure S5.** Retinal gliosis after using Ca^2+^ and Mg^2+^-free BSS as the tamponade agent during surgery. **Table S1.** Summary of details of antibodies and conditions. **Table S2.** Summary of details of *in vivo* ophthalmic follow up of transplanted ESC-RPE grafts in NHPs.

## Data Availability

The data used to support the findings of this study are included within the article.

## Abbreviations

hPSC: Human pluripotent stem cell
AMD: Age-related macular degeneration
RPE: Retinal pigment epithelium
NHP: Non-human primates
hESC: Human embryonic stem cell
hiPSC: Human induced pluripotent stem cell
PET: Polyester-terephthalate
PLGA: Poly(lactic-co-glycolic acid)
XF-Ko-SR medium: Xeno-free differentiation medium containing KnockOut DMEM supplemented with 15% KnockOut SR XenoFree CTS
EB: Embryoid body
TEER: Transepithelial electrical resistance
ZO-1: Zonula occludens-1
Na^+^/K^+^-ATPase: Sodium-potassium adenosine triphosphatase
MERTK: MER Proto-Oncogene, tyrosine Kinase
ELISA: Enzyme-linked immunoassay
PEDF: Pigment epithelium-derived factor
POS: Photoreceptor outer segments
TRITC: Tetramethylrhodamine
IACUC: Institutional Animal Care and Use Committee
ARVO: Association for Research in Vision and Ophthalmology
AAALAC: American Animal Association LAC
miOCT: Microscope-integrated intraoperative optical coherence tomography
IOP: Intraocular pressure
SD-OCT: Spectral domain optical coherence tomography
BAF: Blue laser autofluorescence
IR: Infrared reflectance
FA: Fluorescein angiography
ERG: Electroretinography
ISCEV: Clinical electrophysiology of vision
LA: Light-adapted
DA: Dark-adapted
H&E: Hematoxylin and eosin
ONL: Outer nuclear layer
RT: Room temperature
bRD: Bleb retinal detachment
BSS: Balanced salt solution
PFCL: Perfluorocarbon liquid
ELM: External limiting membrane
GFAP: Glial fibrillary acidic protein
MHC-II: Major histocompatibility complex class II
IFN-γ: Interferon gamma
GMP: Good manufacturing practice
RRD: Rhegmatogenous retinal detachments
