# Supplementary material for: Submacular integration of hESC-RPE monolayer xenografts in a surgical non-human primate model
Source: Stem Cell Res Ther. 2021 Jul 27;12:423. doi: 10.1186/s13287-021-02395-6 (PMC8314642; doi:10.1186/s13287-021-02395-6)
Supplement: Supplementary file 2 — Additional file 1: Figure S1. In vitro characterization of the hESC-RPE cells. Figure S2. Graphical representation of shipment temperatures and times. Figure S3. Postoperative in vivo analysis with multimodal imaging in an animal with unfavorable outcome. Figure S4. Effect of different subretinal BSS injection modes on fluorescein angiography. Figure S5. Retinal gliosis after using Ca2+ and Mg2+-free BSS as the tamponade agent during surgery. Table S1. Summary of details of antibodies and conditions. Table S2. Summary of details of in vivo ophthalmic follow up of transplanted ESC-RPE grafts in NHPs. [file 13287_2021_2395_MOESM1_ESM.docx]

# Supplemental Information

# Supplemental Figures

#### **Figure S1**

**
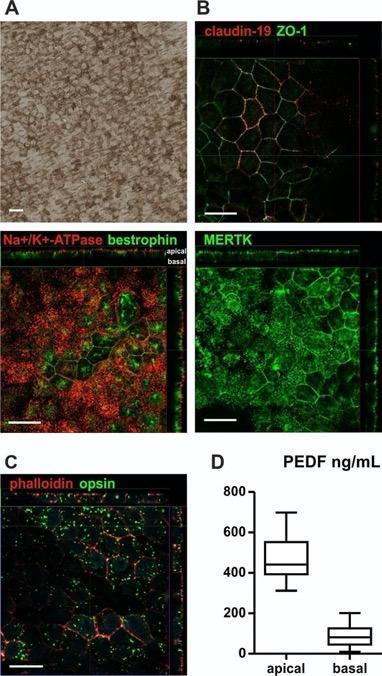
**

*In vitro* characterization of the hESC-RPE cells. **A,** Representative brightfield micrograph shows the morphology and pigmentation of hESC-RPE in culture. **B,** Representative confocal micrographs after immunofluorescence labeling show expression and subcellular polarization of tight junction proteins ZO-1 and claudin-19, Na^+^/K^+^ -ATPase pump, ion channel bestrophin, and phagocytosis receptor MERTK. **C,** Representative confocal micrograph shows the phagocytosis of isolated porcine POS by hESC-RPE *in vitro*. Filamentous actin was visualized with phalloidin. Vertical confocal sections show internalization of POS. Scale bars 20 µm. **D,** Apical and basal PEDF secretion (ng/mL) by hESC-RPE cells grown on PET insert for 50 ± 12 (mean ± SD) days. Secretion was measured from 6 individual experiments (in total 27 inserts). The box plot shows the minimum, first quartile, median, third quartile, and maximum.

#### **Figure S2**

**
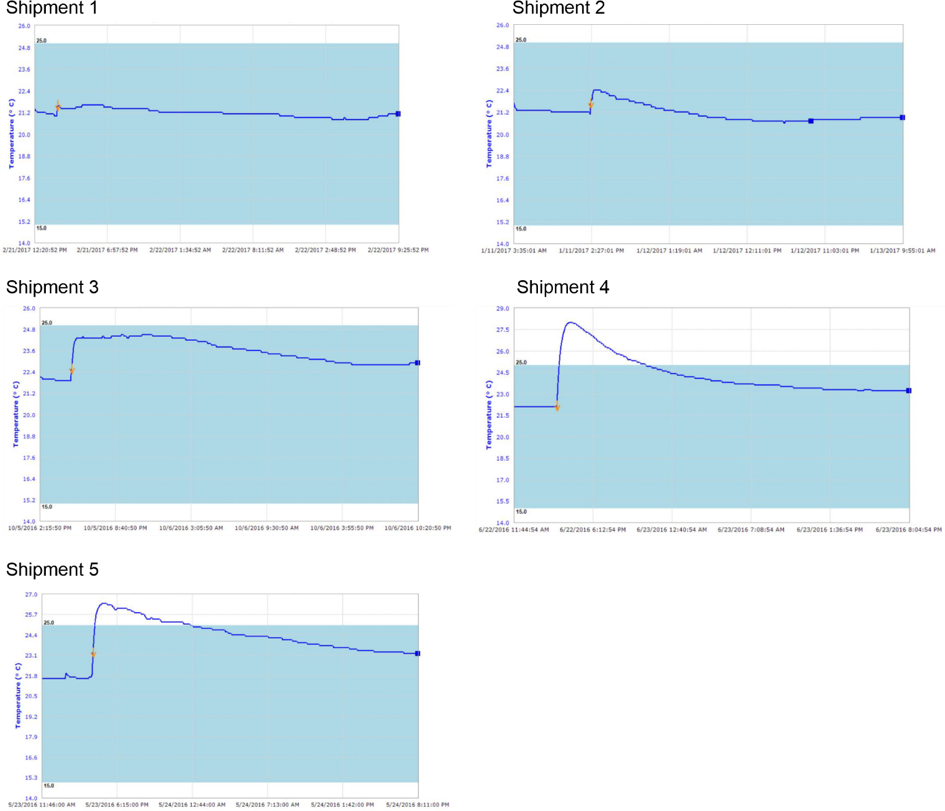
**

Graphical representation of shipment temperatures and times. The low and high extremes of each shipment were 21.6 to 20.8 °C, 20.6 to 22.4 °C, 21.9 to 24.5 °C, 22.1 to 28 °C and 21.6 to 26.4 °C, respectively. The arrow marks the time of adding the cells into the prepared thermo-container.

#### **Figure S3**


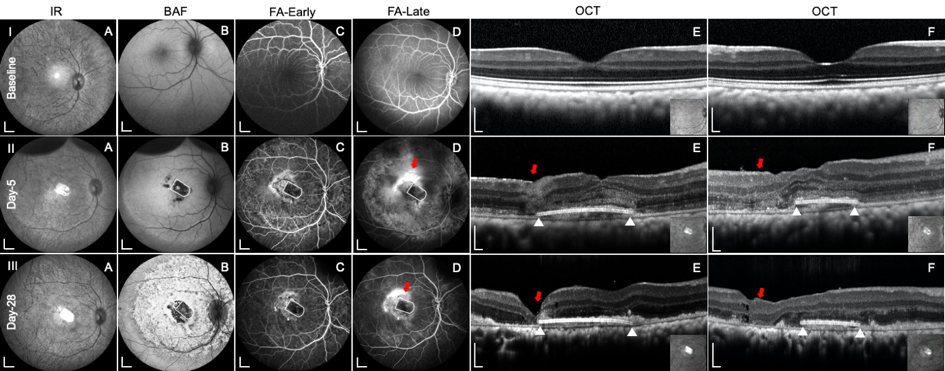


Postoperative *in vivo* analysis with multimodal imaging in an animal with unfavorable outcome. **Line I, II and III,** Representative case of submacular hESC-RPE graft at baseline and Day 5, 28 after surgery. **Column A,** Infrared reflectance (IR) images of posterior fundus. **Column B**, Blue fundus autofluorescence (BAF) images show minor hyper autofluorescence changes at Day 5, that became severe by Day 28. **Column C and D,** Early and late phase fluorescein angiography (FA) shows fluorescein leakage surrounding the hESC-RPE graft (white outlines) in the late phase (red arrows). **Column E and F,** horizontal and vertical SD-OCT scans through the middle of the implants (as shown in inserts). Outer retinal reflectance layers on SD-OCT are grossly preserved both over the hESC-RPE graft (white triangles indicate the edges of the grafts) and the remaining bleb area, except the retinotomy site (red arrows). Scale bars **(A-D)** 2mm, **(E and F)** 200 µm.

#### **Figure S4**


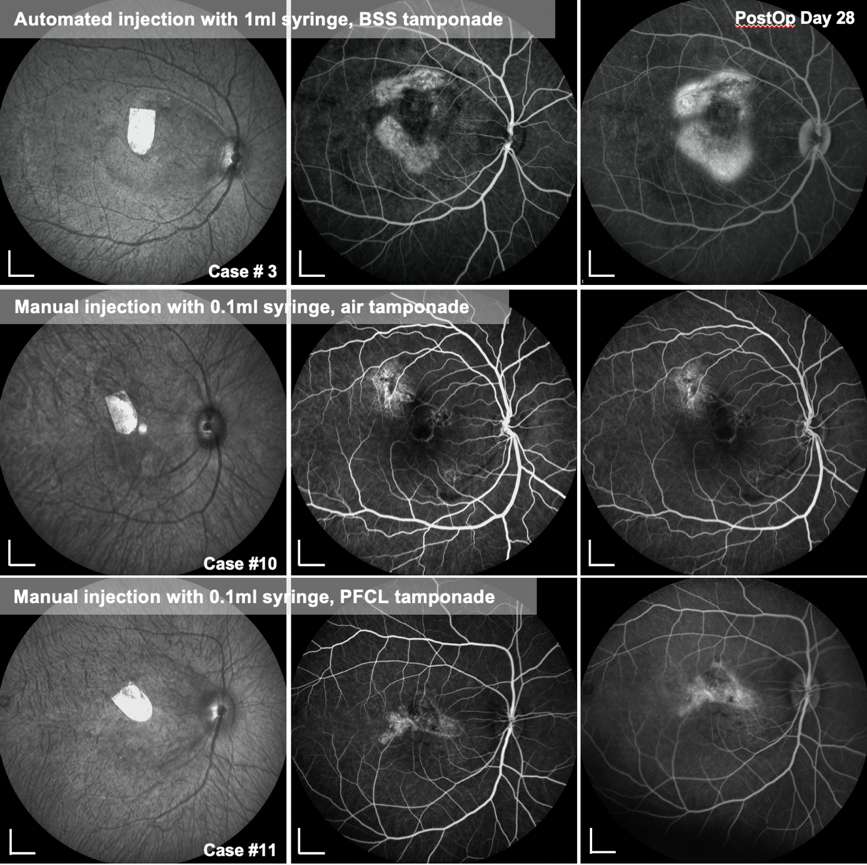


Effect of different subretinal BSS injection modes on fluorescein angiography. The middle panel is early phase FA, the right panel is late phase FA. The left-hand panels show the corresponding infrared reflectance images. An automated, foot pedal-controlled injection with 6-10 psi from the vitrectomy machine produced significant subretinal scarring as evidenced by fluorescein leakage in the late phase (upper row), whilst with a manual injection under air (middle row) or PFCL tamponade (lower row) fluorescein staining is only seen at the site of the former RPE wound. Note that there is no leakage over or immediately surrounding the hESC-RPE implant. All images were taken at post-operative day 28.

#### **Figure S5**
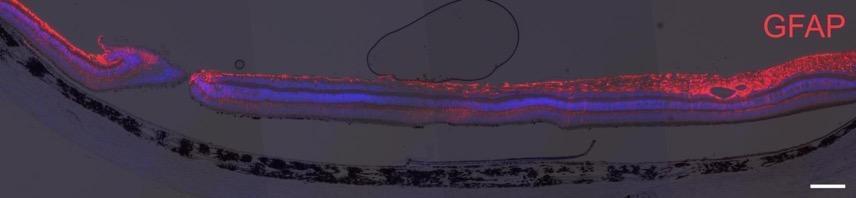


Retinal gliosis after using Ca^2+^ and Mg^2+^-free BSS as the tamponade agent during surgery. Glial cells were visualized by immunostaining for GFAP. Representative image of epifluorescent and brightfield micrograph overlay shows strong upregulation of retinal GFAP. Scale bar, 200 µm.

# Supplemental Tables

## Table S1 Summary of details of antibodies and conditions.

| **Antibody/dye name** | **Manufacturer** | **Cat#** | **Application** | **Prim./sec.** | **Antigen retrieval**  **(paraffin sections)** | **Dilution** |
| --- | --- | --- | --- | --- | --- | --- |
| claudin-19 | R&D Systems | MAB6970 | ICC | Primary |  | 1:50 |
| ZO-1 | Invitrogen | 61-7300 | ICC | Primary |  | 1:100 |
| Na+/K+ -ATPase | Abcam | ab7671 | ICC (paraffin sections) | Primary |  | 1:200 (1:50) |
| bestrophin | Abcam | ab14928 | ICC (paraffin sections) | Primary | 10 mM Sodium Citrate,  0.05% Tween20, pH 6 | 1:100 (1:50) |
| MERTK | Abnova | H00010461-M01 | ICC (paraffin sections) | Primary | 10 mM Sodium Citrate,  0.05% Tween20, pH 6 | 1:50 (1:50) |
| rhodopsin | Sigma-Aldrich | O4886 | ICC | Primary |  | 1:200 |
| TRITC-phalloidin | Sigma-Aldrich | P1951 | ICC | - |  | 1:600 |
| claudin-3 | Thermo Fisher Sci. | 34-1700 | ICC (paraffin sections) | Primary | 10 mM Sodium Citrate,  0.05% Tween20, pH 6 | (1:50) |
| TRA-1-85 | a kind gift from prof. Peter Andrews,  University of Sheffield, UK | - | IHC | Primary | 10 mM Sodium Citrate,  0.05% Tween20, pH 6 | 1:50 |
| STEM121 | Cellartis/Takara | AB-121-U-050/  Y40410 | IHC | Primary | 10 mM Sodium Citrate,  0.05% Tween20, pH 6 | 1:50 |
| rhodopsin | Merck Millipore | MAB5356 | IHC | Primary | 10 mM Sodium Citrate,  0.05% Tween20, pH 6 | 1:100 |
| opsin | Merck Millipore | AB5405 | IHC | Primary | 10 mM Sodium Citrate,  0.05% Tween20, pH 6 | 1:100 |
| GFAP | Merck Millipore | MAB3402 | IHC | Primary | 10 mM Sodium Citrate,  0.05% Tween20, pH 6 | 1:200 |
| vimentin | Nordic BioSite | BSH-7100-1 | IHC | Primary | 10 mM Sodium Citrate,  0.05% Tween20, pH 6 | 1:100 |
| CD3-ε | Santa Cruz Biotech. | sc-1127 | IHC | Primary | 10 mM Sodium Citrate,  0.05% Tween20, pH 6 | 1:100 |
| Iba1 | Abcam | ab5076 | IHC | Primary | 10 mM Sodium Citrate,  0.05% Tween20, pH 6 | 1:100 |
| HLA-DP, DQ, DR | Agilent/Dako | M0775 | IHC | Primary | 10 mM Sodium Citrate,  0.05% Tween20, pH 6 | 1:100 |
| ezrin | Abcam | ab4069 | IHC | Primary | 10 mM Sodium Citrate,  0.05% Tween20, pH 6 | 1:100 |
| A568 anti-mouse IgG | Molecular Probes | A10037 | ICC | Secondary |  | 1:200 |
| A488 anti-rabbit IgG | Molecular Probes | A21206 | ICC | Secondary |  | 1:200 |
| A647 anti-mouse IgG | Invitrogen | A31571 | IHC | Secondary |  | 1:200 |

## Table S2 Summary of details of *in vivo* ophthalmic follow up of transplanted ESC-RPE grafts in NHPs

| **Surgical outcome** | **Case ID** | **Post-operative inflammation** | **RPE defect region (BAF)** | **Vascular leak (FAF at D28)** | **Graft position** | **Subretinal fluid (OCT at D28)** | **Fovea atrophy (OCT at D28)** | **ELM and ONL above the graft (OCT at D28)** |
| --- | --- | --- | --- | --- | --- | --- | --- | --- |
| vorable | #7 | Vitreous clear since D5 | Around the graft and scrape area | No | Stable | No | No, no scar | 3/4 ELM continuous, ONL become slightly thinner |
| Favorable | #8 | Vitreous clear since D5 | Around the graft and scrape area | No | Stable | No | No, tiny scar | ELM continuous, ONL thickness maintained |
| Favorable | #9 | Vitreous clear since D5 | Around the graft and scrape area | No | Stable | No | Yes, obvious | 1/2 ELM continuous, ONL became obvious thinner. |
| Favorable | #10 | Vitreous clear since D5 | Around the graft and scrape area | No | Stable | No | No, tiny scar | ELM continuous, ONL become slightly thinner |
| Favorable | #11 | Vitreous blur but able to capture grafts at D5, clear since D14 | Around the graft and scrape area | No | Stable | No | No, tiny scar | ELM continuous, ONL became obvious thinner. |
| Unfavorable | #3 | Vitreous blur but able to capture grafts at D5, clear since D14 | Within bRD at D5, spread widely to entire AK at D14 and maintained till D28. | Yes | Stable | No | Yes, obvious | 2/3 ELM continuous, ONL became obvious thinner. |
| Unfavorable | #5 | Vitreous clear since D5 | Within bRD at D5, massive defect beyond AK, across optic disc since D14 and progress sever till D28. | Yes | Stable | Minimum under implant | Yes, obvious | ELM discontinuous, ONL became very thin, partially disappear |
| Unfavorable | #6 | Vitreous clear since D5 | Within bRD at D5, spread 1/4 AK till D28. | Yes | Stable | No | No, tiny scar | ELM continuous, ONL become slightly thinner |
| Unfavorable | #12 | Vitreous blur but able to capture grafts at D5, clear since D14 | Massive beyond AK, across optic disc since D5. | No | Stable | Minimum above implant | Yes, obvious | ELM continuous, ONL become obvious thinner. Cell deris can be seen at PRs layer. |
| Unfavorable (transition case) | #13 | Vitreous clear since day 5 | Around the graft and scrape area | No | Stable | Minimum above implant | Yes, obvious | ELM continuous, ONL became obvious thinner. |

# Supplementary Videos

## Video S1

Key steps of submacular transplantation of hESC-RPE graft in a surgical removal naïve RPE model in non-human primates.

1. Create posterior vitreous detachment
2. Detach fovea guided by miOCT
3. Perform retinotomy
4. Scrape naïve RPE
5. Transplant hESC-RPE/ PET graft
6. Adjust position guided by miOCT
7. Drain subretinal fluid under miOCT
